# Prenatal Diagnosis of Persistent Left Superior Vena Cava Raises Suspicion for Coarctation of Aorta

**DOI:** 10.7759/cureus.30220

**Published:** 2022-10-12

**Authors:** Kosuke Yonehara, Kazuya Terada, Mikio Morine

**Affiliations:** 1 Pediatric Cardiology, Shikoku Medical Center for Children and Adults, Zentsuji, JPN; 2 Obstetrics and Gynecology, Shikoku Medical Center for Children and Adults, Zentsuji, JPN

**Keywords:** pediatric clinical cardiology, fetal echocardiography, prenatal diagnosis, coarctation of aorta, persistent left superior vena cava

## Abstract

A pregnant woman was referred to our hospital with a persistent left superior vena cava (PLSVC) and a slightly smaller left heart at 35 weeks of gestation. Fetal echocardiography revealed a small aortic valve and a slightly narrow aortic isthmus. A PLSVC was identified in the dilated coronary sinus. We did not suspect strong coarctation of the aorta (CoA). Echocardiography on postnatal day 0 revealed a slightly narrow isthmus (3.1 mm with a pressure gradient of 5-10 mmHg). When the ductus arteriosus was closed, the isthmus narrowed rapidly to 2.4 mm, and the pressure gradient reached 30 mmHg, which was an indication of surgical arch repair. Aortic arch repair was performed with cardiopulmonary assistance on postnatal day 23. This case presents two lessons. First, when PLSVC is prenatally diagnosed, CoA should be suspected. Second, once CoA is suspected, careful observation of the aortic arch, particularly the isthmus, should be performed.

## Introduction

Persistent left superior vena cava (PLSVC) is among one of the most common congenital abnormalities, with an estimated prevalence of approximately 0.3%-0.5% in the general population [1]. PLSVC has no significant hemodynamic effects because systemic venous blood drains to the right heart in the majority of cases. PLSVC is easily detected using a three-vessel view on fetal echocardiography and is associated with congenital heart disease (87%) and extracardiac anomalies (60%) [2]. Isolated PLSVC is reportedly associated with coarctation of the aorta (CoA) in 21% of patients [3]. Prenatal diagnosis of simple CoA remains challenging because the intracardiac structure tends to be normal, which means that abnormal findings are too poor to be detected during fetal echocardiography screenings. In addition, the aortic arch, particularly the isthmus, is difficult to observe. In some cases, simple CoA may be an emergency that requires surgical repair during the neonatal period. Therefore, a prenatal diagnosis is desirable. Here, we report a case of prenatally diagnosed PLSVC in which simple CoA progressed rapidly after birth.

## Case presentation

**Figure 1:**
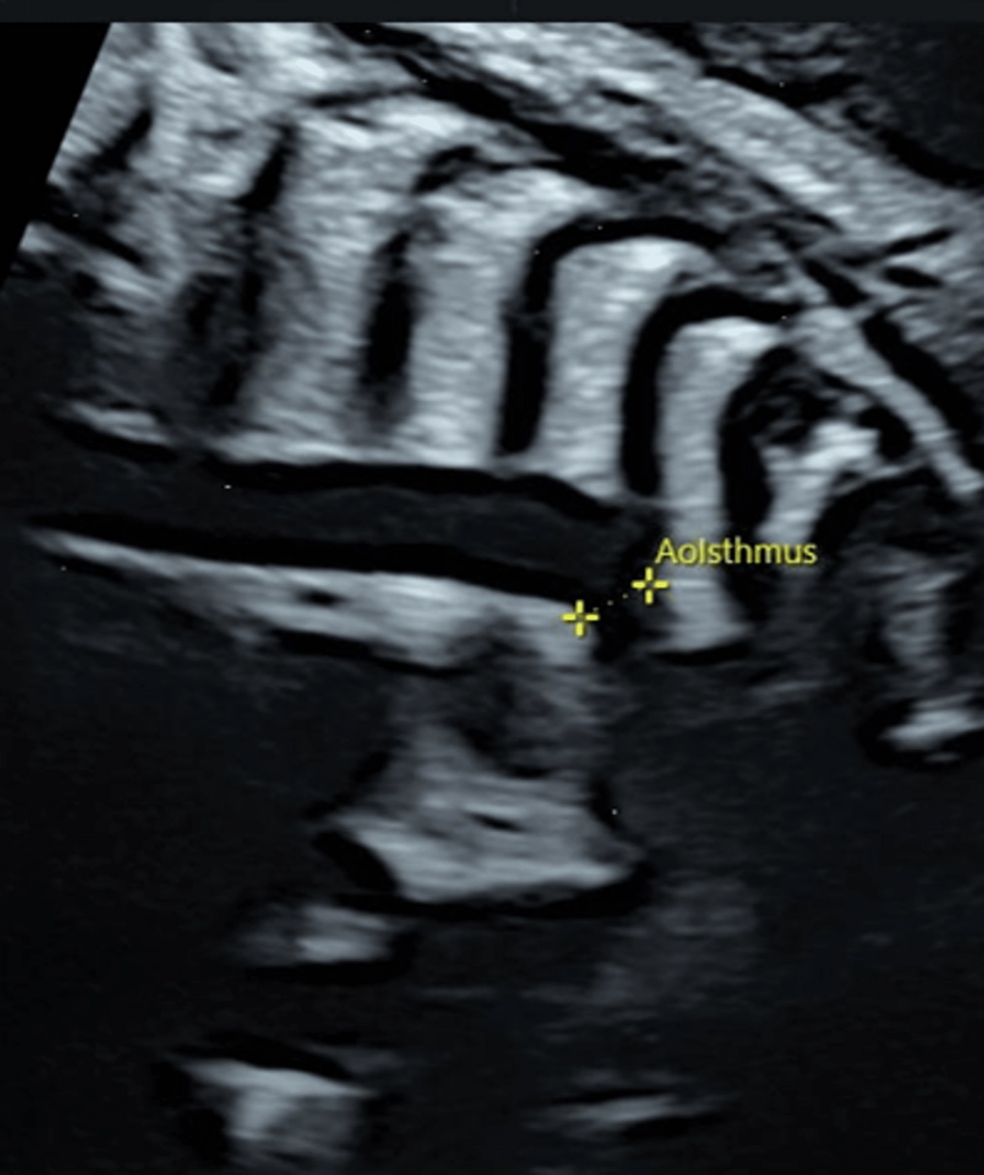
Fetal echocardiography Fetal echocardiography at 35 weeks' gestation revealed aortic isthmus measured in 3.8 mm (z = -2.4).

A 29-year-old Japanese woman in her first pregnancy was referred to our hospital with a PLSVC and a slightly smaller left heart at 35 weeks of gestation. Fetal echocardiography revealed a small left ventricle; the mitral valve diameter was 7.6 mm (z = -2.0), and the tricuspid valve diameter was 11.0 mm (z = -0.14). The size of the aortic valve was 3.9 mm (z = -3.7), and the peak velocity was 92 cm/s, which suggested no stenosis. A slightly narrow aortic isthmus (3.8 mm; z = -2.4) was also observed (Figure 1).

**Figure 2:**
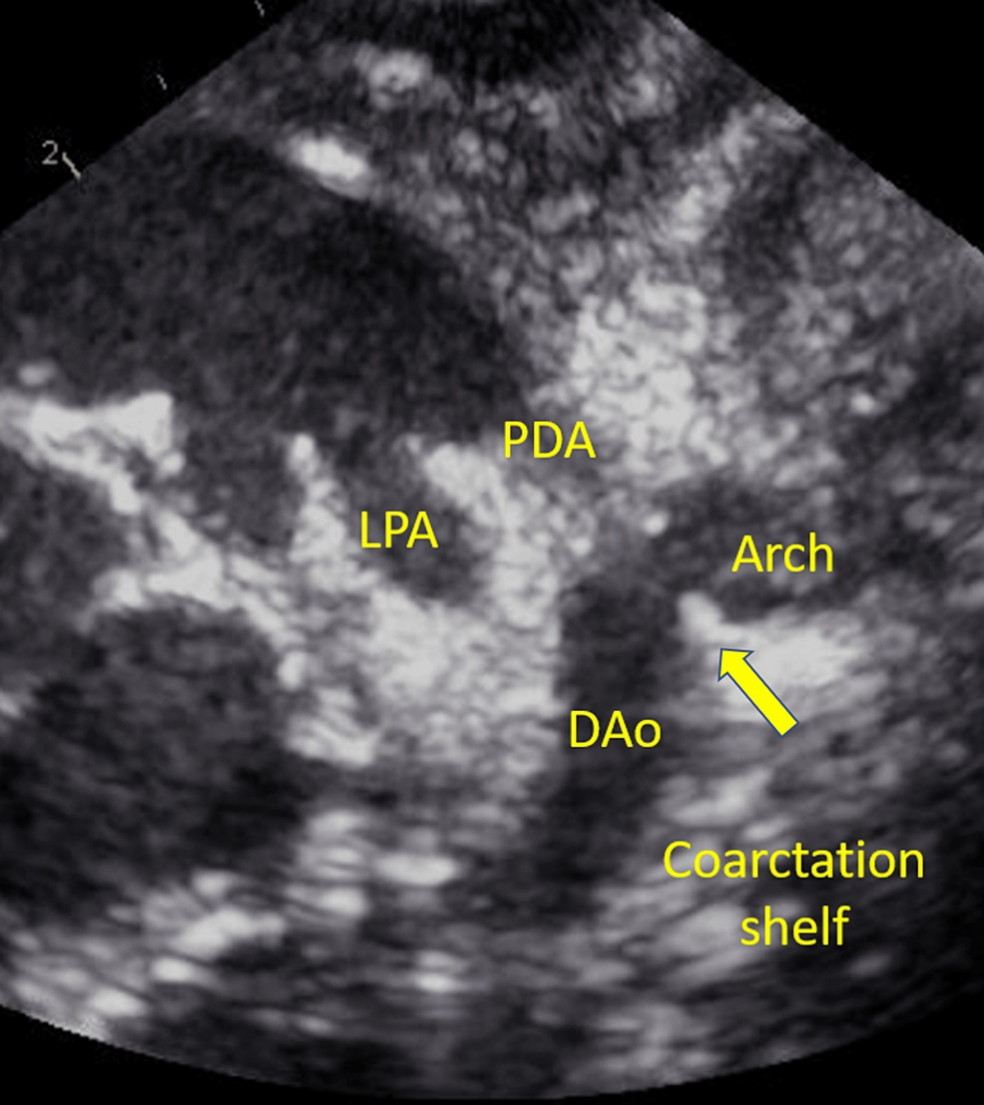
Postnatal echocardiography (arrow) Echocardiography, after DA closed, showed a coarctation shelf on the isthmus. PDA: patent ductus arteriosus, LPA: left pulmonary artery, DAo: descending aorta, DA: ductus arteriosus.

**Figure 3:**
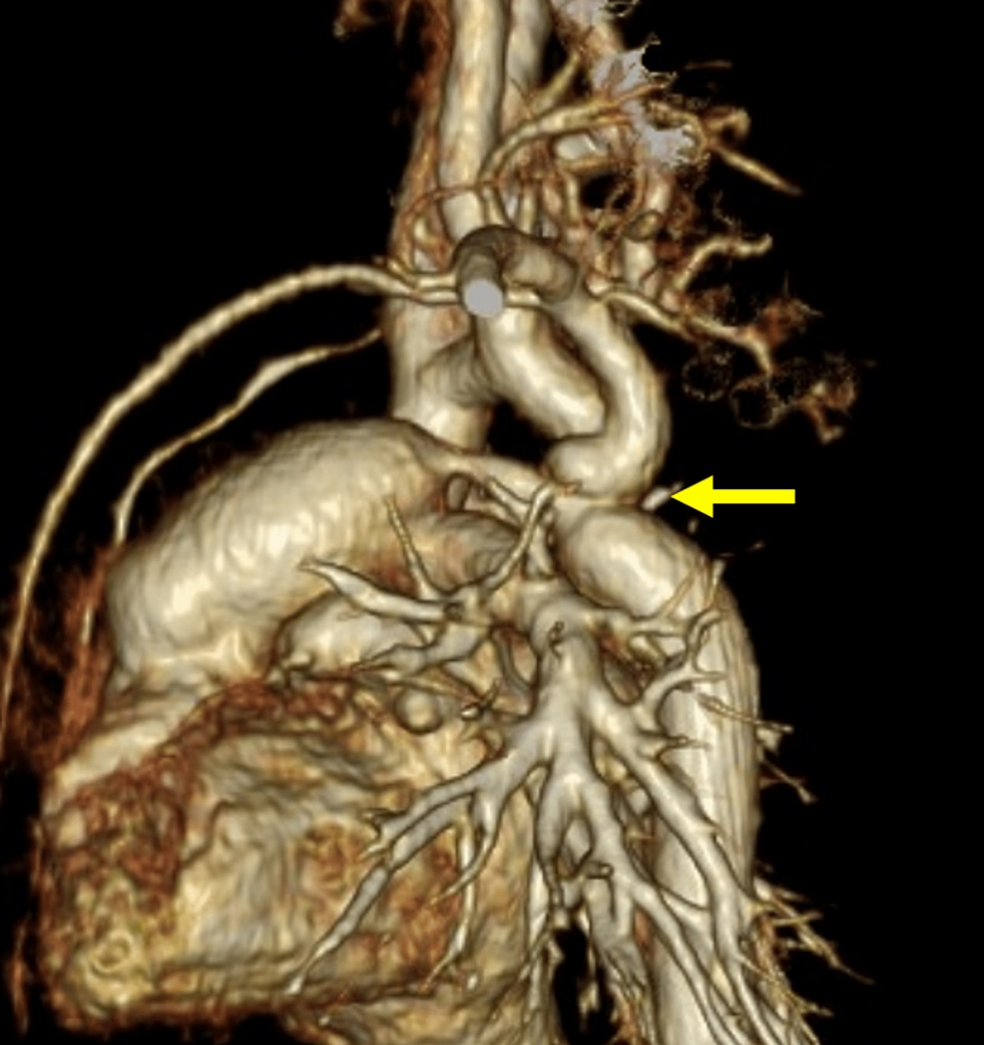
Enhanced computed tomography (arrow) Enhanced computed tomography on postnatal day 4 showed a narrowing isthmus.

A PLSVC was identified in a dilated coronary sinus (CS). No other major anomalies were observed. Follow-up fetal echocardiography at 37 weeks revealed no remarkable changes. Although we did not strongly suspect simple CoA at this point, we planned to assess the neonate for CoA. The neonate was delivered at 40 weeks and two days of gestation with a birth weight of 3,640 g. Apgar score was 8/9 points (1 minute/5 minute). The G band revealed a normal chromosomal type (46 XX). Echocardiography on postnatal day 0 revealed a bicuspid aortic valve with a small diameter (4.8 mm, z = -3.5). The aortic valve showed no regurgitation or stenosis, and the peak velocity was 1.1 m/sec. The PLSVC drained into the right atrium. The mitral valve was 7.9 mm (z = -2.0), which formed a small contrast to the tricuspid valve (12.1 mm, z = -0.5). The mitral valve showed a peak velocity of 1.1 m/sec and a mean pressure gradient of 1.9 mmHg, which suggested no stenosis. Although the isthmus of the aortic arch was slightly narrow (3.1 mm, the pressure gradient between the upper and lower limbs was 5 and 10 mmHg on day 1), the patient was diagnosed with mild CoA, and isthmus size without prostaglandin E1 (PGE1) was observed. The shunt direction of the ductus arteriosus (DA) was continuous left-to-right, suggesting that PGE1 was not required to maintain perfusion of the lower limbs. When the DA was closed, the isthmus of the aortic arch narrowed gradually. On postnatal day 10, the pressure gradient between the upper and lower limbs reached 30 mmHg, indicating the need for surgical repair of the aortic arch. The isthmus diameter decreased to 2.4 mm (Figures 2, 3).

On postnatal day 23, an aortic arch repair by extended ascending aortic anastomosis was performed with cardiopulmonary assistance. The patient’s postoperative course was uneventful, and no pressure gradient was observed. She was discharged from our hospital on postnatal day 47. Outpatient observation of the aortic and mitral valves will continue.

## Discussion

In our case, although CoA was not strongly suspected during the prenatal period, the isthmus narrowing progressed rapidly, requiring neonatal surgical intervention for CoA. This case presents two lessons. First, when PLSVC is prenatally diagnosed, CoA should be suspected. Second, once CoA is suspected, careful observation of the aortic arch, particularly the isthmus, should be performed. The PLSVC drains approximately 20% of the entire venous return; therefore, significantly enhanced venous return via the CS forces an increase in its dimensions. An enlarged CS in the three-vessel view can facilitate prenatal diagnosis of PLSVC.

Tyrak et al. reported that PLSVC is related to a substantial reduction in mitral valve size (2.6 cm^2^; mean, 4.2 ± 1.8 cm^2^), which may be due to the pressure exerted by an enlarged CS on the left atrium and mitral ring. The tricuspid valve area (5.3 cm^2^) did not differ significantly from the average value of 4.8 ± 1.6 cm^2^ [4]. Familiari et al. reported that several parameters, particularly in the left inflow and outflow tracts, differed significantly in fetuses with CoA [5]. In other words, fetuses with PLSVC tend to have small mitral and aortic valves, which are associated with CoA.

In neonates, diagnosis of simple CoA is difficult because of the presence of a patent DA. DA can obscure the anatomy and make it difficult to observe the narrowing of the aortic isthmus [6]. In our case, although the pressure gradient between the upper and lower limbs was initially 5-10 mmHg, it increased to 30 mmHg due to the coarctation shelf as the DA closed. The coarctation shelf is a prominent posterior infolding in the vessel media. The ductal tissue is believed to encircle aortic constriction during ductal closure. The coarctation shelf is more commonly detected postnatally; therefore, prenatal diagnosis is challenging. In addition, the presence of the shelf had high specificity but low sensitivity for CoA, which is explained in part by difficulties in visualization during prenatal echocardiography [5].

## Conclusions

PLSVC is one of the most common congenital anomalies. Although many cases of PLSVC are asymptomatic, it is associated with the sizes of the mitral valve, aortic valve, and aortic arch. It is comparatively easy to detect PLSVC using a three-vessel view on fetal echocardiography. In contrast, CoA is one of the most difficult congenital heart diseases to diagnose prenatally. PLSVC could be an essential marker for prenatally suspected not only extracardiac malformations but also CoA if the left ventricle is small. Once CoA is suspected prenatally, careful postnatal observation of the isthmus and the pressure gradient between the upper and lower limbs should be continued until the DA is closed. Careful follow-up can prevent the occurrence of CoA during the neonatal period.
